# Unveiling potential natural promoters of calcium oxalate kidney stones in the urine via anion-exchange chromatography, crystal assays, and proteomics

**DOI:** 10.1007/s11010-026-05633-8

**Published:** 2026-07-08

**Authors:** Piyaporn Rattananinsruang, Paleerath Peerapen, Somsakul Phuangkham, Sasinun Detsangiamsak, Ekkarin Chotikawanich, Suchai Sritippayawan, Visith Thongboonkerd

**Affiliations:** 1 Medical Proteomics Unit, Research Department, Faculty of Medicine Siriraj Hospital, Mahidol University, Bangkok, 10700 Thailand; 2 Division of Urology, Department of Surgery, Faculty of Medicine Siriraj Hospital, Mahidol University, Bangkok, 10700 Thailand; 3 Division of Nephrology, Department of Medicine, Faculty of Medicine Siriraj Hospital, Mahidol University, Bangkok, 10700 Thailand; 4 Medical Proteomics Unit, Research Department, Siriraj Hospital, Mahidol University, 6th Floor - SiMR Building, 2 Wanglang Road, Bangkoknoi, Bangkok, 10700 Thailand

**Keywords:** Crystal, Mass spectrometry, Nephrolithiasis, Proteome, Stone modulators, Urinary proteins

## Abstract

Knowledge of natural stone promoters in the urine was previously limited, but is essential to develop new targets for better management of calcium oxalate (CaOx) kidney stones. This work, therefore, unveils such information in the urine of CaOx stone formers (patients with stones). Urinary proteins were fractionated by DEAE/GigaQ anion-exchange chromatography, and individual fractions were subjected to multiple crystal assays. The fractions with the summed crystal-promoting score ≥ 3 were subjected to proteomic analysis using nanoLC-ESI-Qq-TOF tandem mass spectrometry (MS/MS). Almost all of the chromatographic fractions (SFQ1-SFQ9) showed promoting effects on CaOx crystallization, growth, aggregation and crystal-cell adhesion. Among them, SFQ2, SFQ8 and SFQ9 provided the greatest summed crystal-promoting score, implicating their roles in stone promotion. MS/MS successfully identified 12, 38 and 6 proteins in fractions SFQ2, SFQ8 and SFQ9, respectively. Among all proteins identified, CD44 antigen, galectin-3-binding protein, kallikrein-1, and protein AMBP were found in more than one fraction, suggesting that they might serve as candidates for the stone promoters. These findings narrow the gap to better understand the pathogenesis and offer opportunities to define new therapeutic targets for better management of CaOx kidney stones.

**Supplementary Information:**

The online version contains supplementary material available at https://doi.org/10.1007/s11010-026-05633-8.

## Introduction

Several attempts have been made to uncover the mystery and mechanisms of kidney stone genesis [1]. Proteomics has been applied to characterize stone matrix constituents that are related to the stone genesis [2–4]. Associations between matrix proteins inside calcium oxalate (CaOx) stones and mechanisms preceding the stone genesis have been documented [4–6]. Some matrix proteins, e.g., uromodulin (also known as Tamm-Horsfall protein), osteopontin, urinary prothrombin fragment 1 and nephrocalcin, have been proven to modulate CaOx stone genesis at various stages [4, 7, 8]. However, proteomic analysis of the stone matrix has some limitations, mostly due to the difficulty in evaluating the entire intact stone, limited sample size, inappropriate stone procurement, and ineffective protein extraction from the stone mass [6].

Human urine, an easily accessible biofluid material, is commonly used for studying a variety of kidney and urinary tract problems [9, 10]. Urine chemistry and profiling of urinary composition also have a wide range of applications to investigate health and disease conditions [11, 12]. Urinary proteomics has become one of the most promising tools due to an enormous amount of information obtained from the proteins excreted through the urinary passage [9, 13, 14]. As such, analysis of the urine from kidney stone formers (SF) (or patients with stones) is extremely helpful for implementing suitable treatment and managing the disease [12]. Therefore, attention has been paid to stone-related proteins in the urine [2, 3]. However, proteins in the urine may or may not get involved in the stone genesis process, as many of them may be the downstream result secondary to the stone (e.g., inflammation, tissue injury), not the upstream etiologic factor [2, 3]. Knowledge of natural stone promoters in the urine that play roles in the stone induction is thus required to foster the development of new targets for better management of CaOx kidney stones.

This study aimed to unveil potential natural stone promoters in the urine of CaOx SF. Urinary proteins were fractionated by DEAE/GigaQ anion-exchange chromatography, and individual fractions were subjected to multiple crystal assays. The ones with the summed crystal-promoting score ≥ 3 were subjected to protein identification by proteomics using nanoLC-ESI-Qq-TOF tandem mass spectrometry (MS/MS).

## Materials and methods

### Collection and preparation of urine samples from stone formers (SF)

Urine samples (random voids) were collected from 30 CaOx SF (aged 30–74 years). Each sample was clarified by centrifugation at 500 g for 10 min and then pooled, dialyzed with deionized water (to desalt), lyophilized, and resuspended in a buffer containing 10 mM Tris–HCl and 50 mM NaCl (pH 7.3).

### Fractionation by DEAE anion-exchange and GigaCap(Q650M) column chromatography

From the above, the samples were incubated with DEAE (Bio-Rad) for 30 min at 25 °C, eluted by a buffer containing 10 mM Tris–HCl and 600 mM NaCl (pH 7.3), dialyzed with deionized water, lyophilized, and resuspended in 15 mM Tris (pH 8.7). The samples were further loaded into GigaCap(Q650M) column (Tosoh Bioscience GmbH) and eluted by a buffer containing 15 mM Tris and 1 M NaCl (pH 8.7) at a 0.5 ml/min flow rate, dialyzed, lyophilized, and resuspended in crystallization buffer containing 10 mM Tris–HCl and 90 mM NaCl (pH 7.4). The concentration of proteins in each chromatographic fraction was measured and finally adjusted to 1 mg/ml across all the samples for crystal assays below.

### Crystallization assay

Crystallization assay was done using an equal volume (4 µl) of proteins (1 mg/ml) from each fraction or crystallization buffer without proteins (control) as described in [15, 16] and Supplementary Methods.

### Crystal growth assay

Crystal growth assay was done using an equal volume (4 µl) of proteins (1 mg/ml) from each fraction or crystallization buffer without proteins (control) as described in [17, 18] and Supplementary Methods.

### Crystal aggregation assay

Crystal aggregation assay was done using an equal volume (4 µl) of proteins (1 mg/ml) from each fraction or crystallization buffer without proteins (control) as described in [19, 20] and Supplementary Methods.

### Crystal-cell adhesion assay

Crystal-adhesion assay was done using an equal volume (4 µl) of proteins (1 mg/ml) from each fraction or crystallization buffer without proteins (control) as described in [21, 22] and Supplementary Methods.

### Scoring of crystal-promoting activities of each protein fraction

A crystal-promoting score of 1 was given to each fraction if it showed a statistically significant promoting effect in each of the crystal assays. By contrast, a negative score (− 1) was given for any statistically significant inhibitory effect. The score of 0 was given if there was no statistically significant effect shown by each fraction in each assay. Finally, the sum of all scores assigned to each fraction across all the assays was calculated.

### Protein identification by tandem mass spectrometry (MS/MS)

Protein fractions with the summed crystal-promoting score ≥ 3 were subjected to protein identification by nanoLC-ESI-Qq-TOF MS/MS as described in [23, 24] and Supplementary Methods.

### Statistical analysis

All experimental data were quantified from 3 independent experiments and are reported as mean ± SEM. Multiple comparisons were performed using ANOVA with Tukey’s post-hoc test. A statistically significant difference was determined when *p*-value < 0.05.

## Results

As the urinary proteome is rather complex, proteins recovered from urine samples of CaOx SF were fractionated via DEAE anion-exchange followed by GigaCap(Q650M) column chromatography. A total of 9 chromatographic fractions, namely SFQ1 to SFQ9, were obtained. These individual fractions were then subjected to a series of CaOx crystal assays to evaluate their modulatory effects on the crystals. In each assay, an equal volume (4 µl) of proteins (1 mg/ml) from each fraction or crystallization buffer without proteins (control) was used for fair comparative analyses across all the samples.

**Fig. 1:**
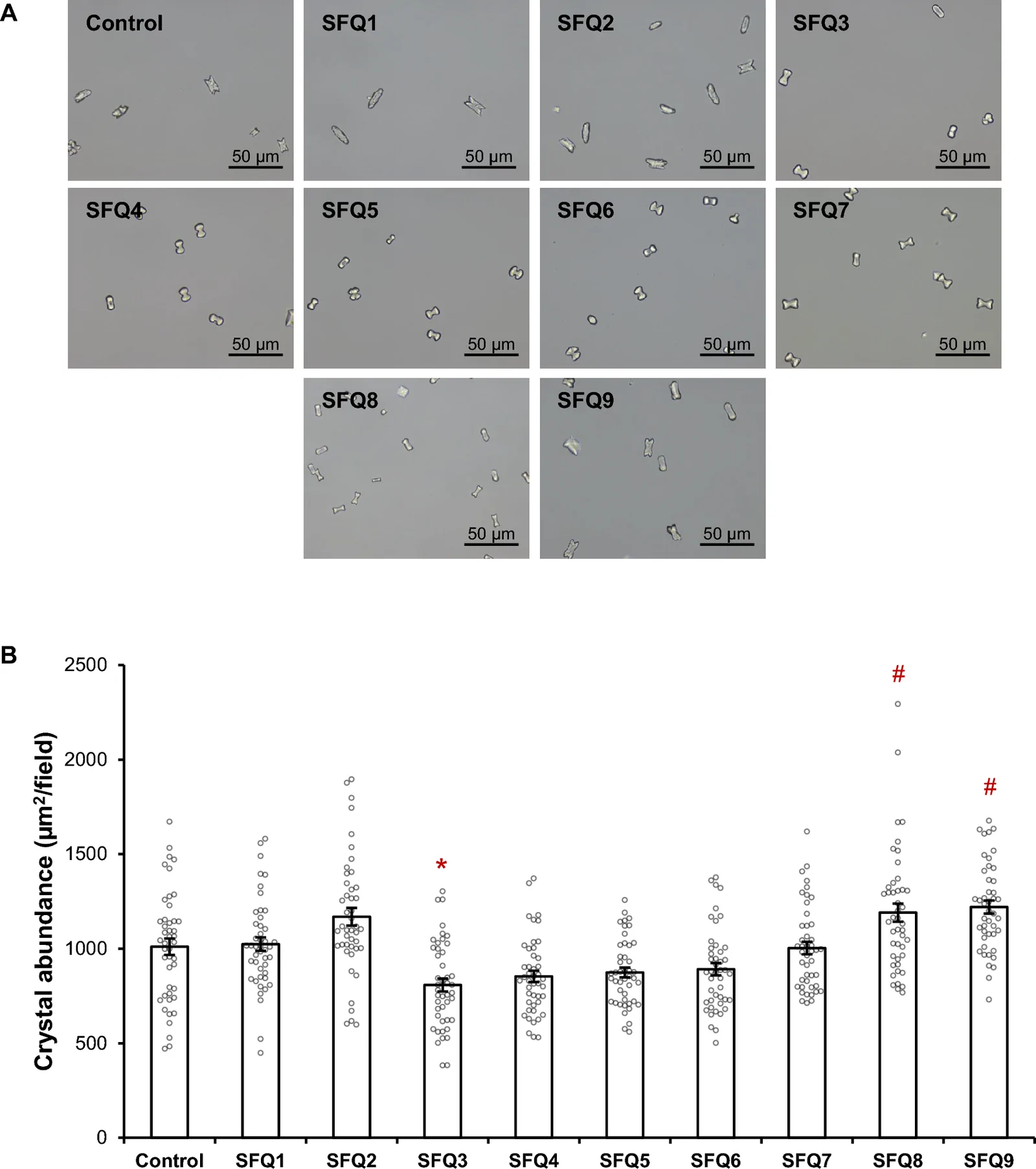
Effects of individual urinary protein fractions (SFQ1-SFQ9) on crystallization. **A**: Crystallization assay was done using an equal volume (4 µl) of proteins (1 mg/ml) from each fraction or crystallization buffer without proteins (control). **B**: Crystal abundance (mean ± SEM) in each sample was measured from ≥ 15 random fields per experiment (× 3 independent experiments) (see Supplementary Methods). # = *p* < 0.05 greater than control (promoting effect); *p < 0.05 less than control (inhibitory effect)

**Fig. 2:**
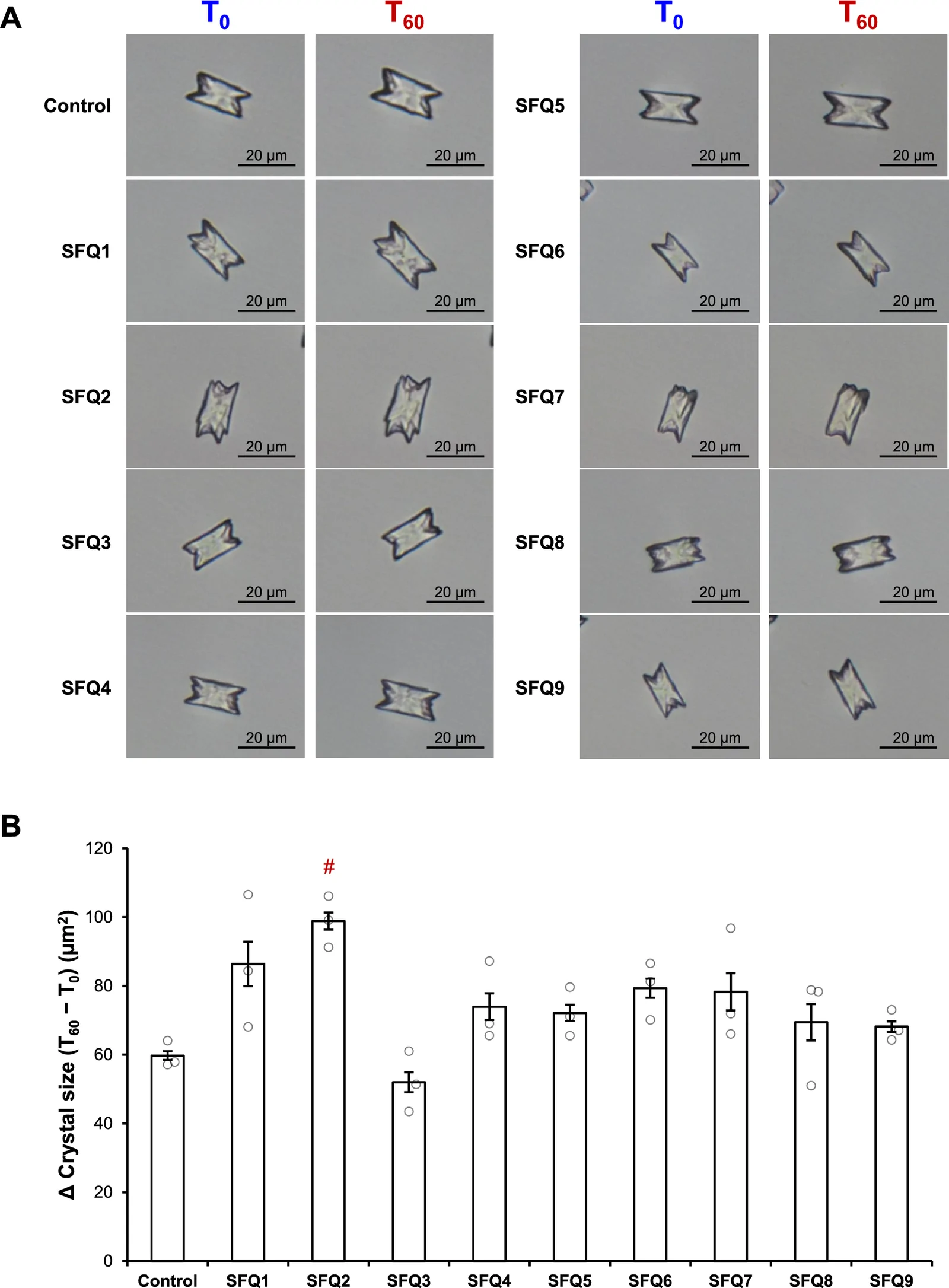
Effects of individual urinary protein fractions (SFQ1-SFQ9) on crystal growth. **A**: Crystal growth assay was done using an equal volume (4 µl) of proteins (1 mg/ml) from each fraction or crystallization buffer without proteins (control). **B**: Δ crystal size (mean ± SEM) representing crystal growth in each sample was averaged from ≥ 100 crystals in 10 random fields per experiment (× 3 independent experiments) (see Supplementary Methods). # = *p* < 0.05 greater than control (promoting effect)

Crystallization assay revealed that the last two fractions (SFQ8 and SFQ9) had a statistically significant promoting effect on CaOx crystallization, as indicated by the increased crystal abundance compared with the control (Fig. 1A and B). By contrast, the fraction SFQ3 showed a modest degree of inhibition on CaOx crystallization, while other fractions showed no statistically significant effects relative to the control (Fig. 1A and B). Crystal growth assay revealed SFQ2 as the only fraction with a statistically significant promoting effect on CaOx crystal growth, as indicated by the increase in Δ crystal size calculated from sizes of the crystals at T_60_ and T_0_ compared with the control, while other fractions showed no statistically significant effects relative to the control (Fig. 2A and B).

**Fig. 3:**
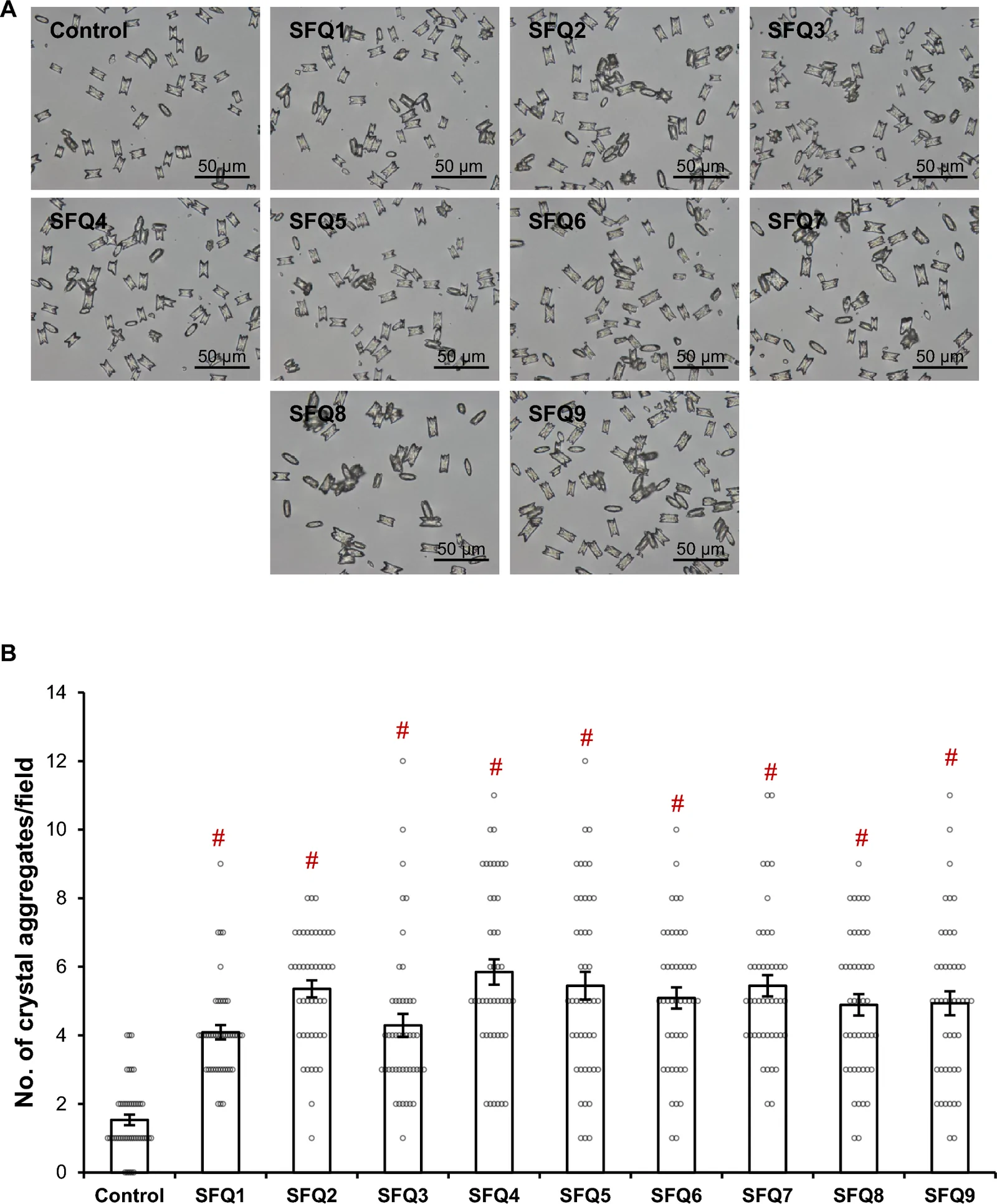
Effects of individual urinary protein fractions (SFQ1-SFQ9) on crystal aggregation. **A**: Crystal aggregation assay was done using an equal volume (4 µl) of proteins (1 mg/ml) from each fraction or crystallization buffer without proteins (control). **B**: The number of crystal aggregates (mean ± SEM) in each sample was counted from ≥ 15 random fields per experiment (× 3 independent experiments) (see Supplementary Methods). # = p < 0.05 greater than control (promoting effect)

**Fig. 4:**
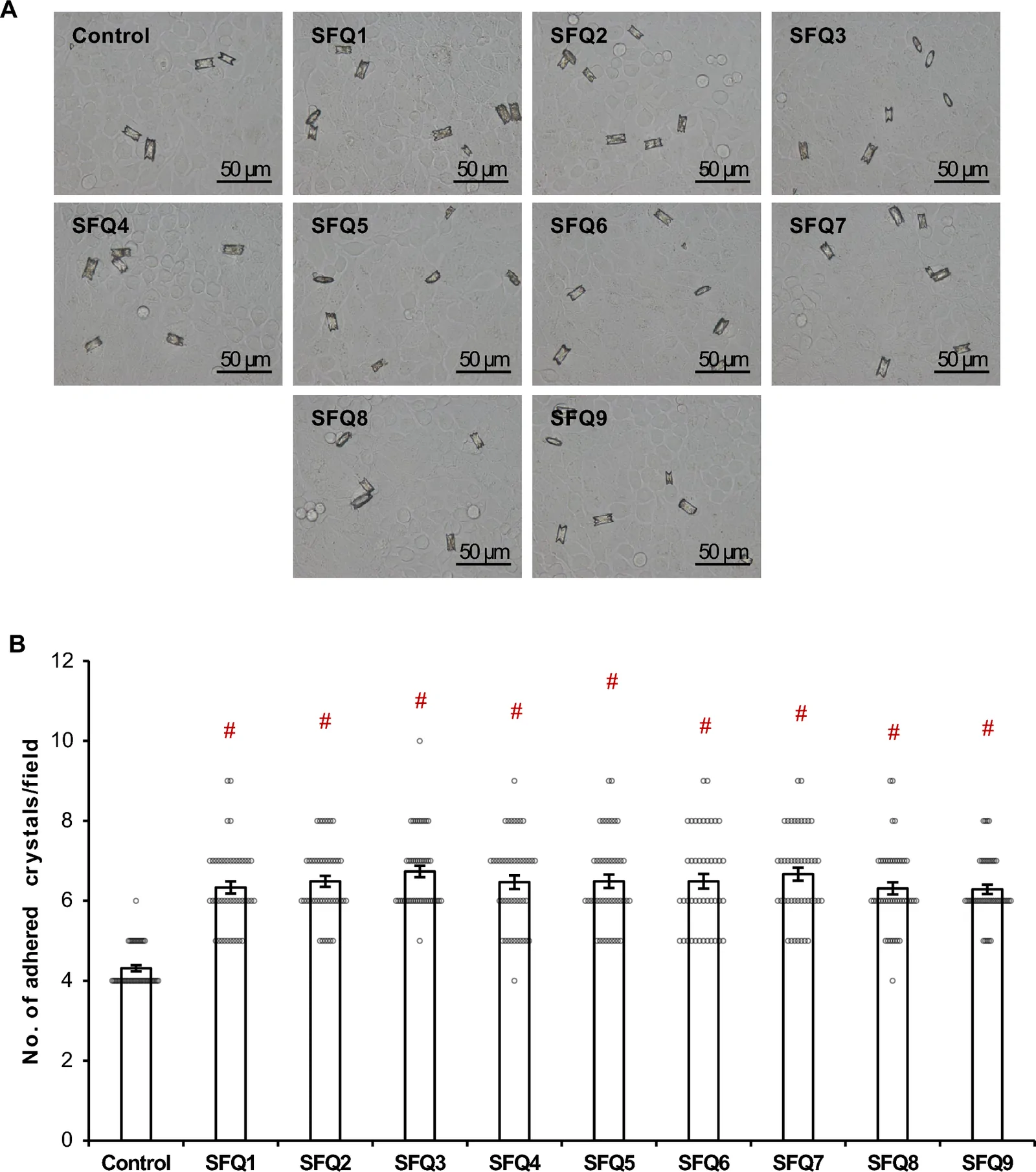
Effects of individual urinary protein fractions (SFQ1-SFQ9) on crystal-cell adhesion. **A**: Crystal-cell adhesion was done using an equal volume (4 µl) of proteins (1 mg/ml) from each fraction or crystallization buffer without proteins (control). **B**: The number of adhered crystals (mean ± SEM) in each sample was counted from ≥ 15 random fields per experiment (× 3 independent experiments) (see Supplementary Methods). # = p < 0.05 greater than control (promoting effect)

Unlike the two former assays, crystal aggregation assay demonstrated that all the chromatographic fractions (SFQ1 to SFQ9) had a statistically significant promoting effect on CaOx crystal aggregation, as indicated by the increased number of crystal aggregates compared with the control (Fig. 3A and B). Similarly, crystal-cell adhesion assay revealed that all the chromatographic fractions (SFQ1 to SFQ9) had a statistically significant promoting effect on CaOx-cell adhesion, as indicated by the increased number of adhered crystals remaining on the cells relative to the control (Fig. 4A and B).

To determine the most promising chromatographic fractions with the most potent promoting effects on CaOx crystals, we assigned a promoting score of 1 to a fraction whenever it showed a statistically significant promoting effect on each of the crystal assays, whereas a score of − 1 was given to the one with an inhibitory effect on each of the crystal assays. Thereafter, all the scores given to each chromatographic fraction were summed across all the crystal assays (Table 1). The ones with the summed crystal-promoting score ≥ 3 were subjected to protein identification by proteomics using nanoLC-ESI-Qq-TOF MS/MS.

**Table 1:** Summary of crystal-promoting activities of each protein fraction

	Crystal-promoting score								
Crystal assay	SFQ1	SFQ2	SFQ3	SFQ4	SFQ5	SFQ6	SFQ7	SFQ8	SFQ9
Crystallization assay	0	0	− 1	0	0	0	0	1	1
Crystal growth assay	0	1	0	0	0	0	0	0	0
Crystal aggregation assay	1	1	1	1	1	1	1	1	1
Crystal-cell adhesion assay	1	1	1	1	1	1	1	1	1
Summ	2	3	1	2	2	2	2	3	3

*1* promote, *− 1* inhibit, *0* no significant modulatory effect

Using this scoring system, SFQ2, SFQ8 and SFQ9 provided the greatest summed crystal-promoting score (Table 1), implicating their roles in stone promotion. NanoLC-ESI-Qq-TOF MS/MS successfully identified 12, 38 and 6 proteins in fractions SFQ2, SFQ8 and SFQ9, respectively (see details in Tables 2, 3 and 4). To further highlight some of these potential stone-promoting urinary proteins, the ones identified from ≥ 2 fractions were then implicated as the candidates of the stone promoters (Table 5). These included CD44 antigen, galectin-3-binding protein (Gal-3BP), kallikrein-1, and protein AMBP (Table 5).

**Table 2:** Summary of urinary proteins identified from SFQ2

Protein name	SwissProt ID	Gene symbol	MS/MS identification score	%Cov	MW (kDa)
[Pyruvate dehydrogenase [acetyl-transferring]]-phosphatase 2, mitochondrial	Q9P2J9	*PDP2*	6.00	1.9	59.98
Collagen alpha-1(XXV) chain	Q9BXS0	*COL25A1*	6.09	2.3	64.77
Ephrin type-A receptor 7	Q15375	*EPHA7*	6.07	1.3	112.10
Eukaryotic translation initiation factor 3 subunit B	P55884	*EIF3B*	6.07	1.6	92.48
Importin subunit alpha-8	A9QM74	*KPNA7*	6.07	2.5	56.94
KN motif and ankyrin repeat domain-containing protein 4	Q5T7N3	*KANK4*	6.50	1.4	107.34
Nuclear protein MDM1	Q8TC05	*MDM1*	6.07	1.8	80.73
PR domain zinc finger protein 13	Q9H4Q3	*PRDM13*	6.13	2.0	73.98
Prosaposin	P07602	*PSAP*	6.12	2.7	58.11
Protein chibby homolog 2	Q8NA61	*CBY2*	6.09	3.3	51.57
Ras-like protein family member 11B	Q9BPW5	*RASL11B*	12.03	2.8	27.51
Rho GTPase-activating protein 39	Q9C0H5	*ARHGAP39*	7.34	0.6	121.28

*%Cov* (number of the identified amino acid residues/total number of amino acid residues in the protein sequence) × 100; *MW* molecular weight

**Table 3:** Summary of urinary proteins identified from SFQ8

Protein name	SwissProt ID	Gene symbol	MS/MS identification score	%Cov	MW (kDa)
Aspartyl/asparaginyl beta-hydroxylase	Q12797	*ASPH*	5.57	1.5	85.86
B-cell receptor-associated protein 29	Q9UHQ4	*BCAP29*	5.54	5.0	28.32
Cartilage oligomeric matrix protein	P49747	*COMP*	5.55	3.8	82.86
CD44 antigen	P16070	*CD44*	13.81	1.1	81.54
Centrosomal protein of 162 kDa	Q5TB80	*CEP162*	5.54	0.5	161.94
Coiled-coil domain-containing protein 121	Q6ZUS5	*CCDC121*	5.54	4.3	33.06
DNA-directed RNA polymerase III subunit RPC1	O14802	*POLR3A*	5.54	0.9	155.64
E3 ubiquitin-protein ligase RNF123	Q5XPI4	*RNF123*	5.59	0.8	148.51
E3 ubiquitin-protein ligase ZNF598	Q86UK7	*ZNF598*	5.55	1.1	98.64
Fibrous sheath-interacting protein 2	Q5CZC0	*FSIP2*	5.65	0.1	780.60
Galectin-3-binding protein	Q08380	*LGALS3BP*	54.73	7.4	65.33
Gamma-aminobutyric acid receptor subunit gamma-1	Q8N1C3	*GABRG1*	5.55	2.2	53.59
Growth/differentiation factor 7	Q7Z4P5	*GDF7*	5.60	2.7	46.95
Immunoglobulin superfamily member 10	Q6WRI0	*IGSF10*	5.62	0.3	290.83
Kallikrein-1	P06870	*KLK1*	43.68	8.8	28.89
Laminin subunit beta-4	A4D0S4	*LAMB4*	5.59	0.7	193.54
Lanosterol synthase	P48449	*LSS*	5.54	2.5	83.31
Large ribosomal subunit protein uL5	P62913	*RPL11*	5.95	3.9	20.25
Multivesicular body subunit 12B	Q9H7P6	*MVB12B*	5.56	3.4	35.62
Nucleotide-binding oligomerization domain-containing protein 1	Q9Y239	*NOD1*	5.55	1.3	107.69
Osteopetrosis-associated transmembrane protein 1	Q86WC4	*OSTM1*	5.93	3.0	37.26
Oxysterol-binding protein-related protein 10	Q9BXB5	*OSBPL10*	5.54	1.6	83.97
P2X purinoceptor 7	Q99572	*P2RX7*	5.69	1.5	68.58
Pepsin A-5	P0DJD9	*PGA5*	10.19	2.8	41.99
Phosphoinositide-3-kinase-interacting protein 1	Q96FE7	*PIK3IP1*	5.72	4.2	28.25
Polyribonucleotide nucleotidyltransferase 1, mitochondrial	Q8TCS8	*PNPT1*	5.61	1.5	85.95
Pro-epidermal growth factor	P01133	*EGF*	5.72	0.6	133.99
Protein AMBP	P02760	*AMBP*	100.68	17.0	39.00
Putative uncharacterized protein FLJ44636	Q6ZTI0		5.54	5.7	12.89
Serine/threonine-protein kinase Nek9	Q8TD19	*NEK9*	5.54	0.7	107.17
Serine/threonine-protein kinase WNK3	Q9BYP7	*WNK3*	10.08	0.4	198.41
Syndecan-4	P31431	*SDC4*	20.14	6.1	21.64
Thrombospondin type-1 domain-containing protein 4	Q6ZMP0	*THSD4*	5.56	1.2	112.45
THUMP domain-containing protein 1	Q9NXG2	*THUMPD1*	5.82	4.5	39.32
Thymus-specific serine protease	Q9NQE7	*PRSS16*	5.59	2.5	55.05
tRNA-dihydrouridine(20) synthase [NAD(P) +]-like	Q9NX74	*DUS2*	5.54	2.4	55.05
Uromodulin	P07911	*UMOD*	16.47	2.8	69.76
WAP four-disulfide core domain protein 2	Q14508	*WFDC2*	20.73	11.3	12.99

*%Cov* (number of the identified amino acid residues/total number of amino acid residues in the protein sequence) × 100; *MW* molecular weight

**Table 4:** Summary of urinary proteins identified from SFQ9

Protein name	SwissProt ID	Gene symbol	MS/MS identification score	%Cov	MW (kDa)
CD44 antigen	P16070	*CD44*	6.88	1.1	81.54
Galectin-3-binding protein	Q08380	*LGALS3BP*	55.55	10.6	65.33
Kallikrein-1	P06870	*KLK1*	8.36	5.0	28.89
Protein AMBP	P02760	*AMBP*	20.01	8.5	39.00
Protein DBF4 homolog B	Q8NFT6	*DBF4B*	6.34	3.4	67.24
Putative uncharacterized protein ZNF252P-AS1	Q0IIN9	*ZNF252P-AS1*	6.35	10.9	22.48

*%Cov* (number of the identified amino acid residues/total number of amino acid residues in the protein sequence) × 100; *MW* molecular weight

**Table 5:** Summary of urinary proteins that may serve as potential CaOx stone promoters

Protein name	SwissProt ID	Gene symbol	Identified from		
			SFQ2	SFQ8	SFQ9
CD44 antigen	P16070	*CD44*		×	×
Galectin-3-binding protein	Q08380	*LGALS3BP*		×	×
Kallikrein-1	P06870	*KLK1*		×	×
Protein AMBP	P02760	*AMBP*		×	×

* ×* fraction in which protein was identified

## Discussion

This study analyzed the protein composition of the SF urine to illustrate the promoting effects of urinary proteins on CaOx crystal parameters. We collected the urine from SF diagnosed with CaOx stones and applied DEAE/GigaCap(Q650M) column chromatography to fractionate urinary proteins to reduce their complexity. The resulting 9 urinary protein fractions (assigned as SFQ1 to SFQ9) were then investigated for their influence in CaOx crystal assays. All the experiments focused mainly on CaOx crystals because CaOx is the major type of kidney stones found in SF [25–28]. From crystallization assay, our results demonstrated a promoting effect of fractions SFQ8 and SFQ9 on CaOx crystal abundance. Another assay revealed that SFQ2 fraction promoted the crystal growth. Moreover, the other two assays showed that all of the urinary protein fractions promoted crystal aggregation and crystal-cell adhesion, indicating that urinary proteins from SF were likely to promote CaOx stone genesis.

Our findings highlight the relevance of interactions between urinary proteins and CaOx crystals in stone genesis. With these interactions to exert stone-promoting activities, these urinary proteins are likely to be incorporated into the CaOx layered structure to develop stone mass [29, 30]. Previous studies have reported that macromolecular mixtures, particularly proteins, isolated from the stone matrix and the urine of SF induce physical aggregation of CaOx crystals [4, 29, 31]. Additional experiments have shown that a mixture of polycationic and polyanionic proteins promotes CaOx crystallization [1, 29, 30]. In concordance, previous investigations have revealed that weakly charged proteins exert modulatory effects on CaOx crystallization [29, 32]. Furthermore, anionic proteins or macromolecules with substantial anionic functionality (e.g., carboxylate group) are thought to promote stone genesis by enhancing CaOx crystal aggregation or adhesion to renal cells [29].

The observations from crystal assays examining chromatographic fractions of SF urinary proteins enlightened that many protein fractions promoted CaOx crystals at multi-step processes involving stone genesis, but at different degrees. We therefore ranked these protein fractions by a scoring system to define the ones with the most potent promoting activities to further characterize proteins in those particular promoting fractions. Having done so, fractions SFQ2, SFQ8 and SFQ9 showed the greatest summed crystal-promoting score, indicating that they should contain several potential CaOx stone-promoting proteins inside. Proteins in these 3 chromatographic protein fractions were then identified by MS/MS analyses, which yielded 12, 38 and 6 proteins in fractions SFQ2, SFQ8 and SFQ9, respectively. As there were many proteins identified, we then pinpointed the ones with the most likelihood of being the CaOx stone-promoting proteins. By combining these results, we found 4 proteins that were identified from at least 2 promoting fractions, implicating their roles as the potential CaOx stone-promoting proteins. These included CD44 antigen, Gal-3BP, kallikrein-1, and protein AMBP.

Renal CaOx crystal deposition correlates with a significant increase in epithelial cell injury, progressive inflammation and regeneration [33–36]. A previous animal study has shown the presence of CD44 in crystal-containing tubules [37]. Another experimental study has reported that CD44 is upregulated when renal cells are exposed to CaOx crystals [38]. It has been evidenced that CD44, in turn, causes crystal retention through its ligands, namely osteopontin and hyaluronan [39, 40]. Not only does the increase in CD44, crystal exposure also upregulates the level of an inflammasome marker (NLR family pyrin domain containing 3) in renal epithelial cells [39, 41]. More importantly, previous evidence has shown that the injured cells have increased crystal-cell adhesion through responsible crystal receptors and cell cycle modifications [42–44]. Taken together, CaOx crystals upregulate CD44 and induce inflammasome activation, leading to tissue injury, which in turn, enhances crystal adhesion and stone genesis.

Roles of Gal-3BP, a 90-kDa soluble secreted protein, have recently been highlighted in various diseases, such as cancer [45, 46], lupus nephritis [47, 48] and infections [49]. Particularly in lupus patients, urinary Gal-3BP is related to renal histological changes [47]. Additionally, urinary Gal-3BP serves as a potential prognostic marker of disease activity of lupus nephritis [47, 48]. The observations also suggest that Gal-3BP may contribute to renal pathologic conditions through proinflammatory mechanisms [47–49]. It is known that damaged renal epithelial cells and the downstream inflammatory response play important roles in kidney stone genesis. Our proteomic findings showing urinary Gal-3BP as a candidate for the potential natural stone promoters in the SF urine were consistent with the previously published data of its presence in CaOx stone matrix [50]. There is evidence illustrating that galectin-3, as a Gal-3BP receptor/ligand, is observed as an indicator of lysosomal membrane damage after CaOx exposure [51]. The accumulation of damaged intracellular organelles causes an impairment of autophagy, resulting in an inflammatory response and oxidative stress [51], both of which can induce kidney stone genesis [52–55].

Kallikrein-1 (KLK1), a serine protease enzyme, is excreted from the kidney into urine and is involved in the generation of kinins. It has been recognized that the kallikrein-kinin system (KKS) displays multiple modes of activation and regulation with physiological and pathophysiological effects. The pathophysiological implications, such as inflammatory response and tissue injury, are likely mediated via KKS activation [56]. Pathogenic mechanisms with respect to crystalline deposits in the kidney can impact renal and systemic disorders such as acute kidney injury (AKI) [57, 58], chronic kidney disease (CKD) [59, 60] and systemic inflammatory response syndrome (SIRS) [61]. According to the occurrence of renal injury and inflammation, crystal-induced cytokine and chemokine secretion should be considered as a mechanism in crystal-induced AKI [33, 62–64]. Consistently, our proteomic experiment highlighted the relevance of KLK1 in the SF urine. Based on the evolving evidence, it seems likely that urinary KLK1 is associated with kidney stone genesis as a disease promoter.

Protein AMBP, a precursor of alpha-1-microglobulin (A1M), is synthesized mainly in the liver and then circulated into the bloodstream [65]. Because A1M-targeting cis elements and transcription factors are responsible for controlling *AMBP* gene expression in the liver, A1M protein is well-suited as the representative to study protein AMBP [65]. In the kidney, A1M protein freely filters through the glomeruli and is reabsorbed by proximal tubules [65]. Increased levels of A1M are linked to various stress conditions [65, 66]. Note that A1M possesses kidney-protective functions to cope with oxidative stress-induced renal damage [65–67]. Although A1M serves as a biomarker for tubular damage, it helps reduce renal injury and recover kidney insufficiency induced by stress [66]. Previously, an upregulation of A1M expression in kidney cells has been described under hyperoxalate state, likely due to its regulatory role against oxalate-induced toxicity [68]. Moreover, the increase in A1M level has been documented in AKI and CKD [69]. Interestingly, other reports have shown the presence of AMBP in CaOx stone matrix [30, 50] and the SF urine [70, 71], consistent with our findings.

**Fig. 5:**
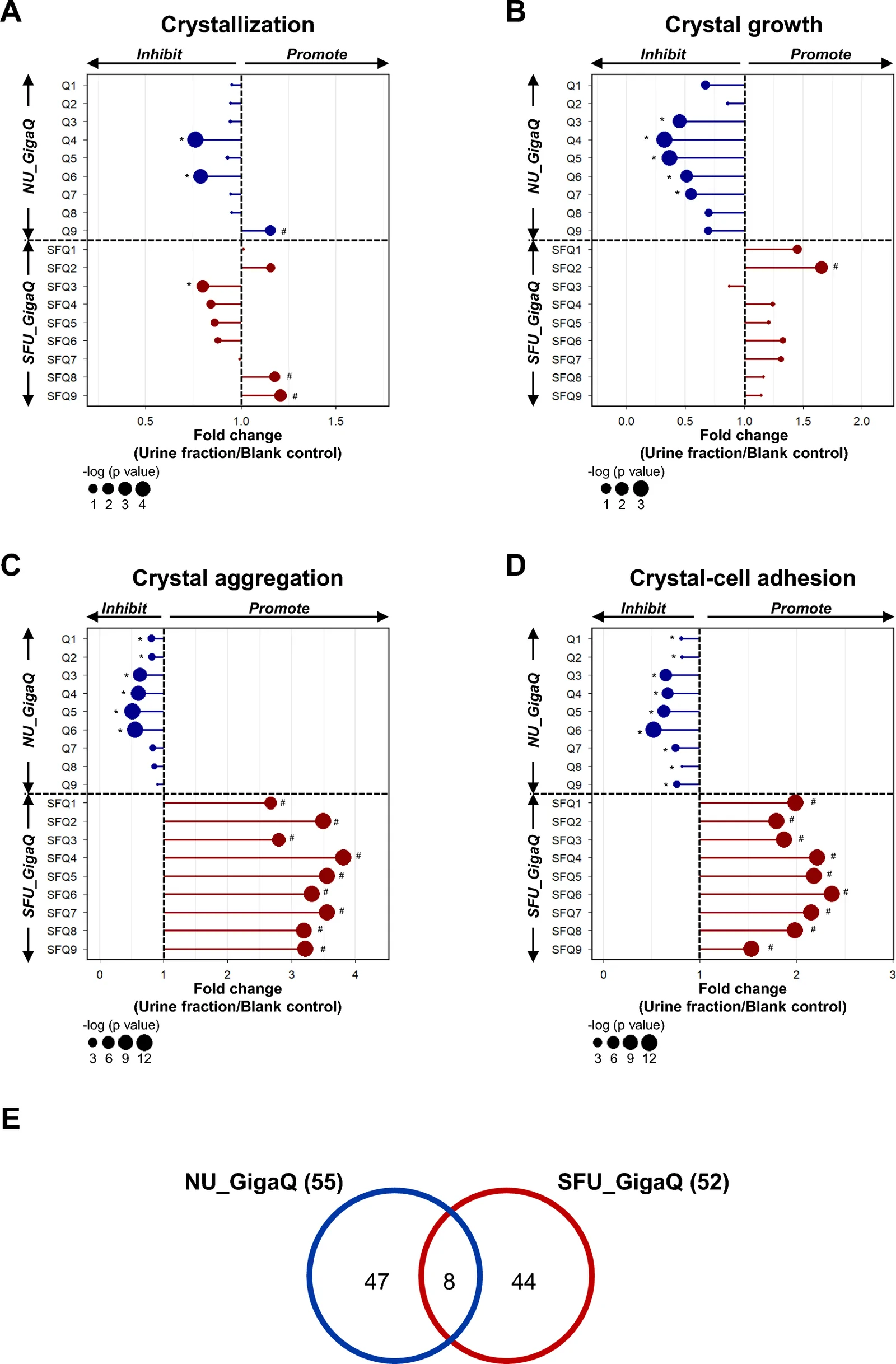
Comparisons of CaOx crystal-modulatory activities of GigaQ-fractionated normal urine (NU_GigaQ) and GigaQ-fractionated SF urine (SFU_GigaQ) and their protein identities. The NU_GigaQ dataset was retrieved from our recent study [72], while the SFU_GigaQ dataset was transferred from Figs. 1, 2, 3 and 4 of the present study. **A**: A lollipop plot of the crystallization-modulatory activity of each protein fraction. Crystallization fold change = Crystal abundance induced by each protein fraction/Crystal abundance under the control condition. **B**: A lollipop plot of the crystal growth-modulatory activity of each protein fraction. Crystal growth fold change = Δ Crystal size induced by each protein fraction/Δ Crystal size under the control condition. **C**: A lollipop plot of the crystal aggregation-modulatory activity of each protein fraction. Crystal aggregation fold change = No. of crystal aggregates induced by each protein fraction/No. of crystal aggregates under the control condition. **D**: A lollipop plot of the crystal-cell adhesion-modulatory activity of each protein fraction. Crystal-cell adhesion fold change = No. of adhered crystals induced by each protein fraction/No. of adhered crystals under the control condition. The fraction with a fold change of > 1 was considered to promote, whereas that with a fold change of < 1 was considered to inhibit CaOx crystals. * = significantly less than the control at p < 0.05, whereas ^#^ = significantly greater than the control at p < 0.05. **E**: The Venn diagram shows the number of proteins identified from the most potent inhibitory fractions in NU_GigaQ and those from the most potent promoting fractions in SFU_GigaQ

It should be noted that we have recently applied anion-exchange chromatography to fractionate proteins from the normal urine obtained from non-stone individuals, aiming to identify natural inhibitors of CaOx stones [72]. We thus compared the present dataset of SF urine using the GigaQ column chromatography (SFU_GigaQ) with the mentioned recent dataset of the normal urine using the same (GigaQ) column chromatography (NU_GigaQ). The crystal assays revealed that almost all of the NU_GigaQ protein fractions had inhibitory effects on CaOx crystallization (Fig. 5A), growth (Fig. 5B), aggregation (Fig. 5C) and crystal-cell adhesion (Fig. 5D). By contrast, almost all of the SFU_GigaQ protein fractions showed promoting effects on CaOx crystallization (Fig. 5A), growth (Fig. 5B), aggregation (Fig. 5C) and crystal-cell adhesion (Fig. 5D). These data are consistent with the recent report illustrating that the whole (unfractionated) urinary proteins from healthy individuals exerted the inhibitory activities to prevent CaOx stones, whereas the whole urinary proteins from SF, on the other hand, promoted CaOx stone formation [55]. More importantly, the MS/MS data showed obviously distinct identities of proteins identified in the urinary fractions with the most potent promoting activities in the present study compared with those with the most potent inhibitory activities in the mentioned recent NU_GigaQ study [72]. There were 44 and 47 unique proteins identified from SFU_GigaQ and NU_GigaQ fractions, respectively, with only 8 common proteins (approximately 8.1%) from a total of 99 being identified from both studies (Fig. 5E), thereby highlighting the contradictory stone-modulatory activities between the SFU_GigaQ and NU_GigaQ fractions.

**Fig. 6:**
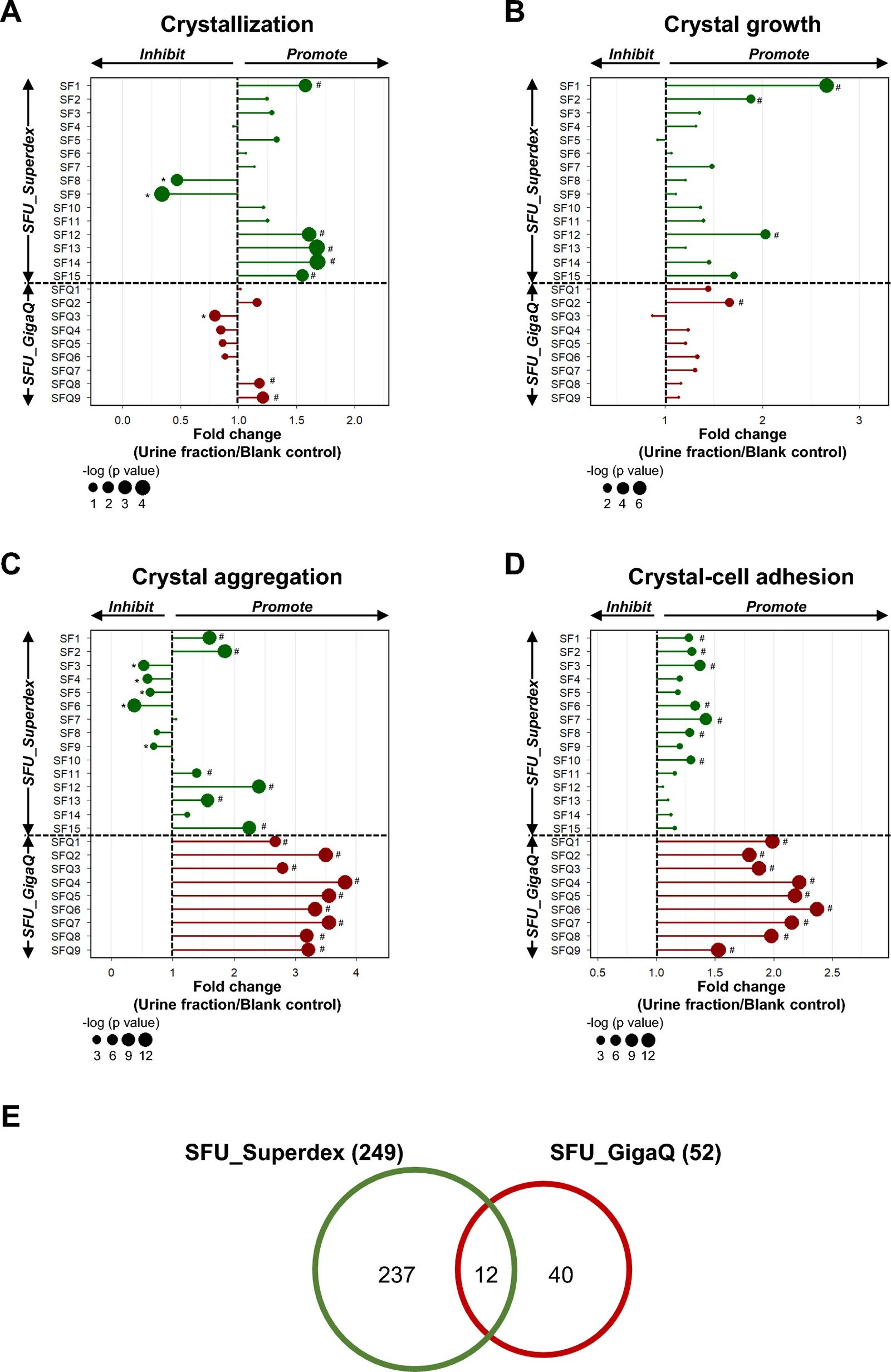
Comparisons of CaOx crystal-modulatory activities of Superdex-fractionated SF urine (SFU_Superdex) and GigaQ-fractionated SF urine (SFU_GigaQ) and their protein identities. The SFU_Superdex dataset was retrieved from our recent study [73], while the SFU_GigaQ dataset was transferred from Figs. 1, 2, 3 and 4 of the present study. **A**: A lollipop plot of the crystallization-modulatory activity of each protein fraction. Crystallization fold change = Crystal abundance induced by each protein fraction/Crystal abundance under the control condition. **B**: A lollipop plot of the crystal growth-modulatory activity of each protein fraction. Crystal growth fold change = Δ Crystal size induced by each protein fraction/Δ Crystal size under the control condition. **C**: A lollipop plot of the crystal aggregation-modulatory activity of each protein fraction. Crystal aggregation fold change = No. of crystal aggregates induced by each protein fraction/No. of crystal aggregates under the control condition. **D**: A lollipop plot of the crystal-cell adhesion-modulatory activity of each protein fraction. Crystal-cell adhesion fold change = No. of adhered crystals induced by each protein fraction/No. of adhered crystals under the control condition. The fraction with a fold change of > 1 was considered to promote, whereas that with a fold change of < 1 was considered to inhibit CaOx crystals. * = significantly less than the control at p < 0.05, whereas ^#^ = significantly greater than the control at p < 0.05. **E**: The Venn diagram shows the number of proteins identified from the most potent promoting fractions in SFU_Superdex and SFU_GigaQ

Indeed, we have also employed a similar (but not identical) approach to identify the candidates of the CaOx stone promoters among many proteins from the SF urine in another recent study [73]. Although DEAE materials were used in both studies to initially enrich anionic proteins, which are the majority of the urinary proteome [74], the present study further fractionated urinary proteins by anion-exchange chromatography using a GigaCap(Q650M) chromatographic column, whereas the mentioned recent study further fractionated urinary proteins by size-exclusion chromatography using a Superdex 200 chromatographic column [73]. The different fractionation methods have allowed us to look at different angles of the urinary proteome, and as a result, the obtained candidates of CaOx stone promoters obviously differed between the two studies. To highlight differences between these two studies on the SF urine, we compared the present dataset of SF urine using the GigaQ anion-exchange column chromatography (SFU_GigaQ) with the mentioned recent dataset of SF urine using the Superdex size-exclusion column chromatography (SFU_Superdex). The crystal assays consistently showed that almost all of the SFU_Superdex and SFU_GigaQ protein fractions had promoting effects on CaOx crystallization (Fig. 6A), growth (Fig. 6B), aggregation (Fig. 6C) and crystal-cell adhesion (Fig. 6D). However, the MS/MS data showed obviously distinct identities of proteins identified in the urinary fractions with the most potent promoting activities in these two studies. There were 40 and 237 unique proteins identified from SFU_GigaQ and SFU_Superdex fractions, respectively, with only 12 common proteins (approximately 4.2%) from a total of 289 being identified from both studies (Fig. 6E), thereby highlighting the different angles of the urinary proteome being analyzed by using different fractionation methods. Combining the results obtained from these two studies would maximize our chance to further characterize the solid natural promoters of CaOx kidney stones.

The benefit of the fractionation strategy has allowed investigations of the complex protein samples more thoroughly, as in our case of the urinary proteome discussed above. More specifically, the fractionation approach prior to analyses by various crystal assays has allowed scoring or prioritizing the urinary protein fractions that should be focused on for subsequent MS/MS identification to narrow down the proteins of interest as the top candidates. Otherwise, too many players also need to be highlighted and are harder to validate. Together with a unique “crystal-promoting score” system implemented for objectively ranking the potency of each fraction, fractions SFQ2, SFQ8 and SFQ9 were identified as the most potent promoting fractions, leading to the identification of 12, 38 and 6 proteins, respectively, within these representative fractions through nanoLC-ESI-Qq-TOF MS/MS. Our study could also pinpoint CD44 antigen, Gal-3BP, KLK1 and protein AMBP as the primary candidates of the stone promoters, as these proteins were identified in multiple potent promoting fractions.

Although with the aforementioned success, a number of limitations in this study should be considered. First, the fractionation method required a large amount of proteins for all CaOx crystal assays and mass spectrometric protein identification. However, the tiny amount of proteins obtained from each SF urine sample prevented us from completing all these experiments. Therefore, pooling individual urine samples was necessary. It is generally known that many factors (e.g., gender, diet, hydration status, exercise, diurnal factor and metabolic status) can cause a wide range of inter-individual variability among urinary protein samples [75–78]. While the pooling strategy made the study feasible, it masks inter-individual differences and prevents assessment of the reproducibility of candidate proteins across patients. Hence, validation of these results in individual SF subjects is required. Second, no direct functional validation was performed on individual candidates of CaOx stone promoters. Therefore, the data remained speculative. Together with a limited number of known stone promoters with experimental evidence [7, 8], validation of these results using purified or recombinant urinary proteins is required to strengthen their roles as the solid stone promoters. Third, although MS/MS protein identification provided sufficient identification scores, some identified proteins had low sequence coverage. Again, validation of the data using purified or recombinant urinary proteins will make these data more solid. Last but not least, the present study did not examine abundance levels of individual proteins identified in each fraction. It would be interesting to analyze the promoting activities of individual candidates relative to their abundance levels that may better reflect their potency.

## Conclusions

Our study has demonstrated the feasibility of a sample preparation method using DEAE/GigaQ anion-exchange chromatography for fractionation of proteins from a complex sample (in our case, the SF urine). We have evaluated the CaOx-promoting profiles from all the eluted fractions by targeting the interaction events in support of kidney stone genesis, such as CaOx crystallization, crystal growth, crystal aggregation, and crystal-cell adhesion. Comparing results from such assays, we selected 3 fractions (namely SFQ2, SFQ8 and SFQ9) that potently promoted CaOx crystals for protein identification. By using nanoLC-ESI-Qq-TOF MS/MS, 12, 38 and 6 proteins were successfully identified from fractions SFQ2, SFQ8 and SFQ9, respectively. Among all proteins identified, CD44 antigen, Gal-3BP, KLK1, and protein AMBP were found in more than one chromatographic fraction, suggesting that they might serve as candidates for the stone promoters. These findings narrow the gap to better understand the pathogenesis and offer opportunities to define new therapeutic targets for better management of CaOx kidney stones.

## Supplementary Information

Below is the link to the electronic supplementary material.Supplementary file1 (PDF 216 KB)

## Data Availability

All data generated or analyzed during this study are included in this published article and are also available from the corresponding author on reasonable request. Additionally, mass spectrometric data have been deposited to the ProteomeXchange Consortium (http://www.proteomexchange.org) via the PRIDE (https://www.ebi.ac.uk/pride) partner repository with the dataset identifier PXD075344 (Username: reviewer_pxd075344@ebi.ac.uk/Pass: 6dXUmoSWb681).
