# Supplementary material for: Unveiling potential natural promoters of calcium oxalate kidney stones in the urine via anion-exchange chromatography, crystal assays, and proteomics
Source: Mol Cell Biochem. 2026 Jul 8;481(8):3887–902. doi: 10.1007/s11010-026-05633-8 (PMC13525045; doi:10.1007/s11010-026-05633-8)
Supplement: Supplementary file 1 [file 11010_2026_5633_MOESM1_ESM.pdf]

Unveiling potential natural promoters of calcium oxalate kidney stones in the urine via anion-exchange chromatography, crystal assays, and proteomics

*Piyaporn Rattananinsruang, Paleerath Peerapen, Somsakul Phuangkham, Sasinun Detsangiamsak, Ekkarin Chotikawanich, Suchai Sritippayawan, and Visith Thongboonkerd**

*Correspondence to: thongboonkerd@dr.com (or) vthongbo@yahoo.com

SUPPLEMENTARY METHODS

Crystallization assay

In brief, 500 µl of 10 mM $CaCl_2 \cdot 2H_2O$ in crystallization buffer (90 mM NaCl in 10 mM Tris, pH 7.4) was put into each well of 24-well plate (Corning Inc.; Corning, NY). Then, 4 µl of 1 µg/µl proteins from each fraction in crystallization buffer or crystallization buffer without any protein (control) was added, followed by 500 µl of 1 mM $Na_2C_2O_4$. The mixture was incubated at 25°C for 60 min, and the resulting CaOx crystals were imaged using an inverted light microscope (Eclipse Ti-S) (Nikon; Tokyo, Japan). Crystal size was measured from at least 15 random fields per sample using NIS-element D software version 4.11 (Nikon). Crystal abundance was then calculated as follows.

(Formula 1)

Crystal abundance (µm²/field) = ∑Crystal sizes (µm²) of all crystals in each field

$$\text{Crystal abundance } (\mu m^2/\text{field}) = \sum \text{Crystal sizes } (\mu m^2) \text{ of all crystals in each field}$$

Crystal growth assay

In brief, 500 µl of 10 mM $CaCl_2 \cdot 2H_2O$ in crystallization buffer was put into each well of 24-well plate, followed by 500 µl of 1 mM $Na_2C_2O_4$. The mixture was incubated at 25°C for 60 min. At this time-point (T_0), 4 µl of 1 µg/µl proteins from each fraction in crystallization buffer or crystallization buffer without any protein (control) was added, followed by another incubation at 25°C for 60 min (T_{60}). At T_0 and T_{60}, CaOx crystals were imaged using an inverted light microscope (Eclipse Ti-S). Crystal size was measured from at least 100 crystals in 10 random fields per sample using NIS-element D software version 4.11. Δ crystal size representing crystal growth was calculated as follows.

(Formula 2)

$$\Delta \text{ Crystal size in each sample } (\mu m^2) = \text{Average crystal size in each sample at } T_{60} (\mu m^2) - \text{Average crystal size in each sample at } T_0 (\mu m^2)$$

Crystal aggregation assay

In brief, 10 mM $CaCl_2 \cdot 2H_2O$ was mixed 1:1 (v/v) with 1 mM $Na_2C_2O_4$, and the mixture was incubated overnight at 25°C. The resulting CaOx crystals were harvested by centrifugation at 2,000 *g* for 5 min, washed with methanol thrice and air-dried overnight at 25°C. An equal amount of CaOx crystals (1,000 µg) was suspended in 1 ml of crystallization buffer in each well of 6-well plate (Corning Inc.), followed by 4 µl of 1 µg/µl proteins from each fraction in crystallization buffer or crystallization buffer without any protein (control). The plate was continuously shaken in a shaking incubator (Zhicheng; Shanghai, China) for 1 h at 150 rpm and 25°C. The resulting crystal aggregates (each has been defined as "an assembly of three or more individual CaOx crystals that tightly joined together") were imaged using an inverted light microscope (Eclipse Ti-S). The number of crystal aggregates was counted from at least 15 random fields per sample.

Crystal-cell adhesion assay

In brief, CaOx crystals were prepared and harvested as described in the crystal aggregation assay above. After air drying, the crystals were decontaminated by UV light radiation for 30 min. MDCK renal cells (ATCC; Manassas, VA) were seeded and grown in each well of 6-well plate (2×10^5 cells/well) for 48 h to obtain a confluent monolayer. The culture supernatant was replaced with the fresh medium containing CaOx crystals (100 µg/ml) and 4 µl of 1 µg/µl proteins from each fraction in crystallization buffer or crystallization buffer without any protein (control). The cells were further incubated for 1 h, followed by vigorous washes with PBS to remove the non-adhered crystals. The remaining (adhered) crystals on the cell surface were imaged using an inverted light microscope (Eclipse Ti-S) and counted from at least 15 random fields per sample.

In-solution tryptic digestion by filter-aided sample preparation (FASP) method

The protein sample with an equal amount (10 µg each) was subjected to buffer exchange into SDT lysis buffer (4% SDS, 100 mM DTT, and 100 mM Tris-HCl; pH 7.6) and reduced by heating at 95°C for 5 min. After cooling down at 25°C, the sample was transferred to an Omega Nanosep 10K device (Pall Corporation; Port Washington, NY), added with 200 µl of 8 M urea in 100 mM Tris-HCl (pH 8.5), and then centrifuged at 14,000 *g* and 25°C for 15 min. This buffer exchange step was repeated one more cycle. The recovered proteins were then alkylated with 100 µl of 50 mM iodoacetamide in 8 M urea/100 mM Tris-HCl (pH 8.5) at 25°C in the dark using ThermoMixer C (Eppendorf; Hauppauge, NY) for 20 min. Thereafter, buffer exchange was performed twice by centrifugation at 14,000 *g* and 25°C for 15 min each using 200 µl of 8 M urea/100 mM Tris-HCl (pH 8.5). The proteins were finally exchanged into 50 mM NH_4HCO_3 and digested with sequencing grade modified trypsin (Promega; Madison, WI) in 50 mM NH_4HCO_3 at a ratio of 1:50 (w/w) trypsin/protein at 37°C for 16-18 h in ThermoMixer C. The digested peptides were collected by transferring the filter unit to a new collection tube and centrifuged at 14,000 *g* and 25°C for 10-20 min. Trypsin activity was then stopped by adding 10 µl of 5% formic acid in 80% acetonitrile (ACN), and the digested peptides were dried by a SpeedVac concentrator (Savant; Holbrook, NY). The peptides were finally resuspended in 0.1% formic acid prior to MS/MS analysis.

NanoLC-ESI-Qq-TOF MS/MS

Separation of the digested peptides was performed using EASY-nLC II (Thermo Scientific; Waltham, MA). Briefly, peptides were loaded from a cooled (7°C) autosampler into an in-house, 3-cm-long pre-column containing 5-µm C18 resin (Dr.Maisch GmbH; Ammerbuch, Germany) and then to an in-house, 10-cm-long analytical column packed with 3-µm C18 resin (Dr.Maisch GmbH) using mobile phase A (0.1% formic acid). The peptides were then separated by mobile phase B (ACN/0.1% formic acid) gradient elution (3-35%) for 150 min at a flow rate of 300 nl/min. Peptide sequences were then analyzed by an ultra-high resolution Qq-TOF MS/MS system (maXis Impact, Bruker Daltonics) in positive mode with ESI nanosprayer ion source. The nanoLC and Qq-TOF MS/MS systems were controlled by HyStar Version 3.2 (Bruker Daltonics) and otofControl Version 4.1 (Bruker Daltonics), respectively. A capillary voltage and spray shield voltage were set at 5,000V and 500 V, respectively. Nebulizer gas was set at 5.0 psi and dry gas flow rate was at 4.0 l/min, 150°C.

For MS scanning, precursor ions were scanned from 50 to 2,200 *m/z* range (resolution = 40,000 at 622 *m/z*) and acquired at 2 Hz (0.5 s total accumulation). For MS/MS experiment, the three most intense precursor ions for every MS scan were selected for further fragmentation. Collision-induced dissociation (CID) MS/MS acquisition was performed at 2 Hz (0.5 s total accumulation, if precursor $\leq 1 \times 10^4$ ion counts) and 10 Hz (0.1 s total accumulation, if precursor $\geq 5 \times 10^5$ ion counts) on the same mass range and resolution set for MS scanning,

whereas singly charged ions were excluded. Smart exclusion parameters were set to minimize repeated acquisitions of the same intense precursor ions (repeated count was 2, dynamic exclusion was set at 0.50 min).

Protein identification

Each MS raw file (.d) was separately analyzed by MaxQuant software package (version 2.1.4.0) (*https://www.maxquant.org*) with built-in Andromeda search engine. Proteins were identified by searching against the UniProtKB/Swiss-Prot human protein database together with commonly observed contaminants and reversed sequences (decoy database) for all entries. The parameters used for protein identification were set as follows: fixed modification = carbamidomethylation (C), variable modifications = oxidation (M) and acetyl (N-terminus), maximal number of missed cleavages = 1, minimum peptide length = 7 amino acids, enzyme = trypsin, first search precursor tolerance = 40 ppm, main search precursor tolerance = 10 ppm, and MS/MS tolerance of time-of-flight (TOF) scans = 20 ppm. Protein false discovery rate (FDR) and peptide-to-spectrum match (PSM) FDR were set at 0.01 calculated based on the search against reversed sequence decoy database.
